# Executive functions in research and practice: a multimethod review of behavioral, subjective, and neurobiological assessment tools

**DOI:** 10.3389/fpsyg.2026.1713652

**Published:** 2026-03-03

**Authors:** Athina Pitta

**Affiliations:** Department of Midwifery, School of Health Sciences, University of Western Macedonia, Ptolemaida, Greece

**Keywords:** assessment tools, computerized testing, ecological validity, executive functions, neuroimaging, performance-based tests, rating scales

## Abstract

Executive functions (EFs) are higher-order cognitive control processes that enable goal-directed behavior, self-regulation, and adaptive functioning across the lifespan. A widely adopted theoretical framework conceptualizes core executive functions as inhibitory control, updating of working memory representations, and cognitive flexibility, rather than treating working memory as an executive function per se. Despite extensive research, the accurate assessment of executive functions remains a major challenge due to their multidimensional and context-dependent nature. This narrative review, informed by systematic search principles, synthesizes peer-reviewed literature published between 2000 and 2024 on executive function assessment. Searches were conducted in Scopus, PubMed, JSTOR, Web of Science, Google Scholar, and HEAL-Link. A total of 85 sources were screened, with 12 representative empirical studies and reviews selected for synthesis. Four major assessment approaches were identified: performance-based tests, computerized assessments, rating scales, and neuroimaging techniques. Each approach offers unique strengths, but also significant limitations related to ecological validity, subjectivity, accessibility, and cost. No single method adequately captures the complexity of executive functioning. The review argues for a multimethod assessment framework integrating behavioral, subjective, and neurobiological measures, and highlights the need for cross-cultural validation, digital innovation, and early identification to improve research and applied practice.

## 1 Introduction

Executive functions (EFs) refer to a set of higher-order cognitive control processes that regulate thoughts, emotions, and actions in the service of goal-directed behavior (Diamond, 2013). Among the most influential conceptualizations is the model proposed by Miyake et al. (2000), which distinguishes three core but interrelated executive functions: inhibitory control, updating of working memory representations, and cognitive flexibility. Importantly, within this framework, working memory is not considered an executive function; rather, executive processes are those that regulate and update the contents of working memory via the central executive system, as originally described by Baddeley and Hitch (1974).

Inhibitory control involves the ability to suppress dominant or automatic responses and regulate behavior in accordance with task demands and social norms. Updating refers to the active monitoring and manipulation of information held in working memory, allowing relevant representations to be maintained while irrelevant information is discarded. Cognitive flexibility denotes the capacity to shift between tasks, mental sets, or strategies in response to changing environmental demands. Higher-order executive abilities—such as planning, problem solving, and decision-making—are thought to emerge from the interaction of these core components (Diamond, 2013; Chan et al., 2008).

Executive functions play a central role in academic achievement, occupational performance, emotional regulation, and psychological well-being. Deficits in EF are implicated in numerous clinical and developmental conditions, including attention-deficit/hyperactivity disorder, traumatic brain injury, and neurodegenerative disorders (Jurado and Rosselli, 2007; Toplak et al., 2013). Consequently, the assessment of executive functions has become a critical concern in neuropsychology, education, and mental health practice.

Despite the proliferation of assessment tools, substantial debate persists regarding whether different methods capture the same underlying constructs and how well they reflect real-world functioning. Performance-based tests, rating scales, computerized tasks, and neuroimaging techniques often yield weak to moderate convergence, raising questions about construct validity and ecological relevance (Toplak et al., 2013). Furthermore, many existing reviews have focused on specific populations (e.g., children) or single assessment modalities, limiting their generalizability.

The present review aims to:

- critically examine the main approaches to assessing executive functions across the lifespan;
- evaluate their strengths and limitations with respect to ecological validity, standardization, and construct overlap; and
- Highlight the value of multimethod assessment strategies that integrate behavioral, subjective, and neurobiological perspectives.

Executive functions develop gradually across the lifespan, showing rapid growth during early childhood, continued refinement through adolescence, and vulnerability to decline in older adulthood (Best and Miller, 2010). These developmental trajectories have important implications for assessment, as executive demands and behavioral manifestations vary substantially across age groups. Nevertheless, many existing reviews have focused on restricted developmental periods, particularly early childhood or aging, limiting the generalizability of their conclusions (Isquith et al., 2005). A lifespan-oriented perspective is therefore essential for understanding both the strengths and limitations of different executive function assessment approaches.

By synthesizing literature published between 2000 and 2024, this review seeks to extend previous work by offering a comprehensive and integrative overview of executive function assessment.

## 2 Materials and methods

This article constitutes a narrative review informed by systematic search principles. Although elements of systematic searching were employed to enhance transparency and coverage, the review does not meet the formal criteria for a systematic review or an umbrella review. Prior reviews and meta-analyses were included to contextualize empirical findings rather than to provide second-level quantitative synthesis.

### 2.1 Search strategy

Relevant studies were identified through searches of Scopus, PubMed, JSTOR, Web of Science, Google Scholar, and the Greek academic repository HEAL-Link. The search period spanned from 2000 to 2024 to capture both foundational and contemporary research. Keywords were used in English and Greek and included combinations of: “*executive functions,” “assessment,” “measurement,” “evaluation,” “inhibitory control,” “updating,” “cognitive flexibility,” “planning,” “rating scales,” “computerized assessment,”* and *“neuroimaging.”* Boolean operators (AND/OR) were applied to refine results.

The exact database-specific search strings, including Boolean operators, are provided in Supplementary Material A to ensure full transparency and replicability.

### 2.2 Inclusion and exclusion criteria

Inclusion criteria were:

- peer-reviewed empirical studies, reviews, or meta-analyses;
- focus on at least one method of executive function assessment; and
- relevance to clinical, educational, or developmental contexts.

Exclusion criteria included conference abstracts, non-peer-reviewed reports, and studies lacking sufficient methodological detail.

### 2.3 Study evaluation variables

The selected studies were analyzed according to the following variables:

1. Executive function component assessed (inhibition, updating, flexibility);
2. Assessment modality (performance-based, computerized, rating scale, neuroimaging);
3. Population and age range;
4. Ecological validity;
5. Psychometric properties;
6. Cultural and contextual applicability.

These variables were chosen to allow systematic comparison across heterogeneous assessment approaches.

### 2.4 Study selection procedure

The study selection process was guided by PRISMA 2020 principles (Page et al., 2021) to enhance transparency while remaining consistent with the scope of a narrative mini-review. Database searches identified 85 records, all of which were screened by title and abstract, as no duplicate records were detected. During this screening stage, 60 records were excluded due to lack of relevance to executive function assessment or failure to meet inclusion criteria.

The remaining 25 reports were retrieved and assessed for eligibility at the full-text level. Of these, 13 reports were excluded for the following reasons: (1) the study did not focus on executive function assessment (*n* = 5), (2) insufficient methodological detail on assessment tools (*n* = 4), or (3) redundancy or substantial overlap with previously published reviews (*n* = 4).

**Figure 1:**
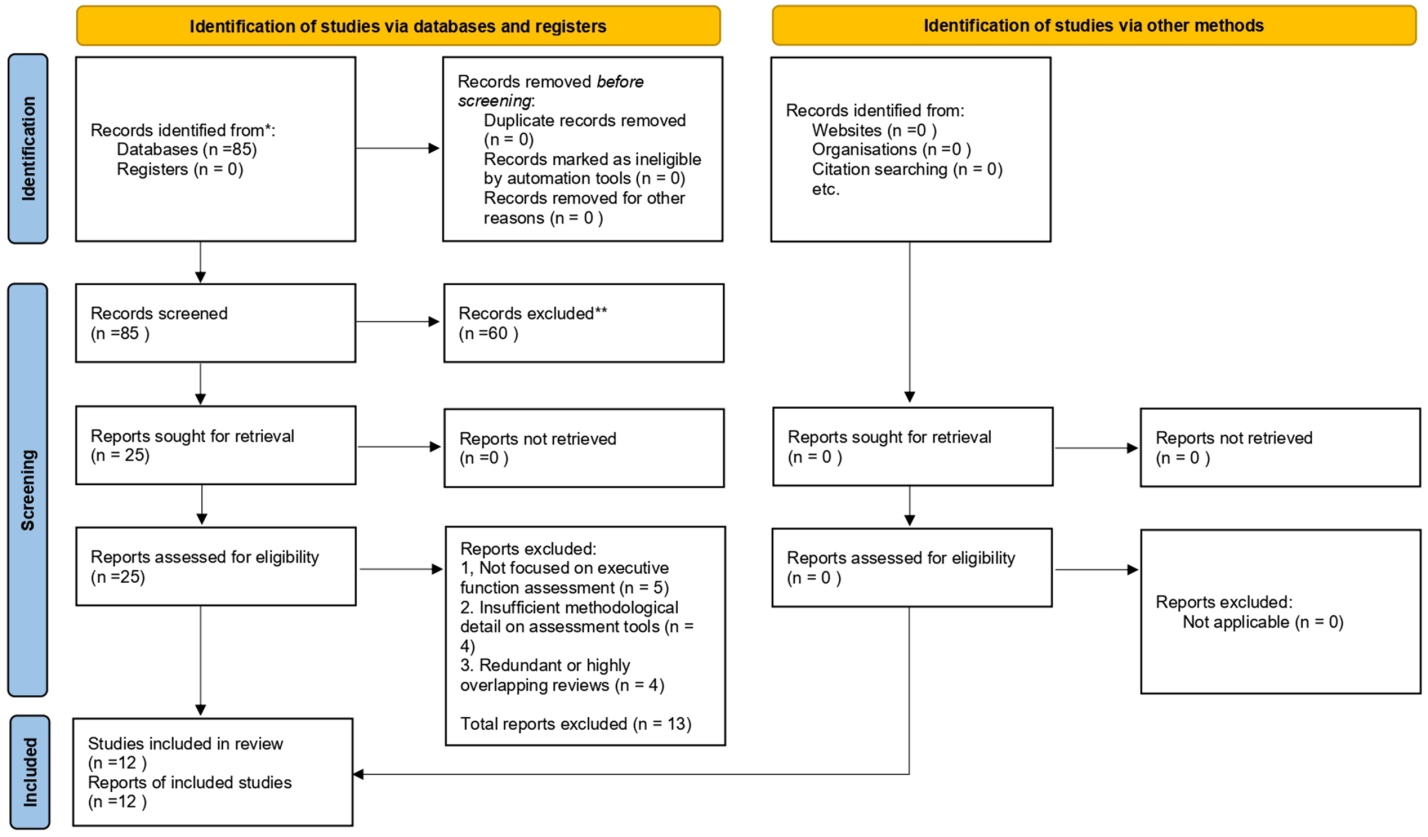
PRISMA 2020 flow diagram illustrating the study selection process. Eighty-five records were identified through database searches. After screening, 60 records were excluded. Twenty-five full-text reports were assessed for eligibility, and 13 were excluded for specified reasons, resulting in 12 studies included in the qualitative synthesis.

A total of 12 studies met the eligibility criteria and were included in the qualitative synthesis. The full study selection process is illustrated in the PRISMA 2020 flow diagram (Figure 1).

## 3 Results: methods for assessing executive functions

Table 1 provides an overview of the main assessment approaches, typical instruments, strengths, and limitations (Souissi et al., 2022). The findings are organized into four thematic categories.

**Table 1:** Executive function assessment approaches: instruments, targeted components, age range, and key characteristics.

Assessment approach	Representative instruments	EF components targeted	Typical age range	Procedure/format	Ecological validity	Key limitations
Performance-based tests	WCST, Stroop, Go/No-Go, Flanker, Trail Making Test, Tower of London, Verbal Fluency	Inhibition, cognitive flexibility, updating	Childhood–older adulthood	Structured laboratory tasks; accuracy and reaction time measures	Low–moderate	Limited ecological validity; influenced by education and culture; assess optimal rather than typical performance
Computerized assessments	SART, CBAAD, VR-based EF tasks	Inhibition, sustained attention, updating	Adolescence–adulthood (increasingly children)	Computerized or immersive tasks with automated scoring	Moderate–high	Require digital literacy; limited normative data; high cost
Rating scales (self/informant)	BRIEF (parent, teacher, self-report), EFI	Behavioral regulation, emotional control, planning	Preschool–older adulthood	Questionnaires completed in everyday contexts	High	Subjectivity; rater bias; limited sensitivity to cognitive processes
Neuroimaging techniques	fMRI, PET, EEG	Neural correlates of inhibition, updating, flexibility	Adolescence–older adulthood	Brain imaging during EF tasks or rest	Low	High cost; low accessibility; indirect behavioral relevance

### 3.1 Performance-based tests

Performance-based tests are the most traditional and widely used tools for assessing executive functions. These standardized tasks typically measure accuracy, reaction time, and cognitive efficiency under controlled conditions (Chan et al., 2008; Toplak et al., 2013; Souissi et al., 2022). Common examples include the Wisconsin Card Sorting Test, Stroop Test, Go/No-Go tasks, Trail Making Test, Tower of London, and verbal fluency tasks.

A key advantage of performance-based tests is their objectivity and standardization, which support reliability and comparability across studies. They are particularly useful for detecting executive dysfunction in clinical populations and for tracking cognitive decline across aging (Faria et al., 2015). However, these tests often suffer from low ecological validity, as performance in structured laboratory settings may not reflect everyday executive functioning. Cultural, educational, and socioeconomic factors may also influence outcomes, limiting generalizability.

### 3.2 Computerized assessments

Computerized assessments, including virtual reality–based tasks and three-dimensional testing environments, have emerged as innovative tools for executive function evaluation (Malegiannaki et al., 2019; Parsons, 2015). These approaches offer precise measurement of reaction times, automated scoring, and adaptive task difficulty, reducing examiner bias and participant fatigue.

Enhanced ecological validity is a notable strength, particularly when tasks simulate real-life scenarios. Nevertheless, computerized assessments require technological infrastructure, digital literacy, and normative data, which constrain their accessibility and clinical utility. High costs and limited cross-cultural validation further restrict widespread application.

### 3.3 Rating scales

Rating scales assess executive functioning through self-reports or informant reports, capturing behavior in everyday contexts (Soto et al., 2020). Instruments such as the Behavior Rating Inventory of Executive Function (BRIEF) and the Executive Function Index (EFI) are widely used across age groups (Isquith et al., 2013).

Rating scales demonstrate high ecological validity (Souissi et al., 2022) and practical relevance, especially for children and adolescents. However, they are inherently subjective and susceptible to rater bias, social desirability, and contextual variability. Importantly, they often show weak correlations with performance-based measures, suggesting that they assess complementary but distinct aspects of executive functioning (Toplak et al., 2013).

### 3.4 Neuroimaging techniques

Neuroimaging methods—including functional magnetic resonance imaging, positron emission tomography, and electroencephalography—provide insights into the neural correlates of executive functions (Alvarez and Emory, 2006; Nowrangi et al., 2014). These techniques have identified consistent involvement of prefrontal and associated networks in executive control processes.

Despite their theoretical value, neuroimaging methods are costly, technically demanding, and characterized by low ecological validity. As such, they are primarily suited for research rather than routine clinical assessment and must be interpreted alongside behavioral measures.

## 4 Discussion

Given the breadth and conceptual complexity of executive functions, the present article was intentionally designed as a narrative mini-review. Rather than providing exhaustive coverage of all available instruments, the review prioritizes conceptual integration, theoretical coherence, and comparative analysis of major assessment approaches across the lifespan. This approach allows for focused discussion of methodological trade-offs and practical implications while remaining consistent with the scope of a mini-review format.

The findings reveal persistent trade-offs between methodological rigor and ecological validity in executive function assessment. Performance-based tests offer precision and standardization but often fail to capture everyday functioning. Rating scales provide ecologically valid insights yet are limited by subjectivity. Computerized assessments and neuroimaging contribute technological and biological depth but face accessibility and practicality challenges.

Concerns regarding the ecological validity of traditional executive function tests are long-standing. Burgess et al. (1998) demonstrated that performance on standardized executive tasks often shows limited correspondence with real-world functioning, particularly in unstructured or novel situations. This discrepancy suggests that laboratory-based assessments may underestimate everyday executive difficulties, especially in individuals who perform adequately under highly controlled conditions. Such findings underscore the importance of complementing performance-based tests with ecologically oriented measures when evaluating executive functioning.

Importantly, several studies have examined the relationship between different assessment methods. Evidence suggests modest correlations between performance-based tests and rating scales, indicating partial overlap but also substantial divergence (Toplak et al., 2013). These findings imply that executive functions are context-dependent and cannot be fully captured by a single method. Rather than reflecting measurement failure, low convergence may indicate that different tools assess distinct dimensions of executive functioning—optimal performance versus typical behavior.

Differences between assessment methods may also reflect the distinction between “cool” and “hot” executive functions. Cool executive functions involve abstract, decontextualized problem solving and are typically assessed using performance-based tasks, whereas hot executive functions are engaged in emotionally salient or motivationally significant contexts (Zelazo and Carlson, 2012). Rating scales, which capture behavior in real-life situations, may therefore be more sensitive to hot executive processes, while laboratory tasks primarily assess cool executive control. This distinction provides a theoretical explanation for the modest correlations often observed between different assessment systems and further supports the need for integrative, context-sensitive evaluation strategies.

Collectively, the literature supports a multimethod assessment framework. Integrating objective tests, subjective reports, and neurobiological data enhances construct validity and provides a more comprehensive profile of executive functioning (Diamond, 2013; Isquith et al., 2013).

## 5 Conclusions and future directions

This review highlights that no single assessment method is sufficient to capture the full complexity of executive functions. Although each approach provides valuable insights, all are inherently limited when used in isolation. Consequently, a multimethod framework that integrates complementary sources of information is essential for accurate assessment, diagnosis, and intervention planning.

Future assessment models should explicitly consider developmental stage (Best and Miller, 2010), prioritize ecological validity (Burgess et al., 1998), and account for contextual demands on executive control, including the distinction between hot and cool executive functions (Zelazo and Carlson, 2012). In parallel, future research should emphasize cross-cultural validation, longitudinal study designs, and the development of integrative assessment frameworks. While digital innovations offer promising opportunities to enhance ecological validity, their implementation must be supported by robust normative data. Finally, early identification of executive function difficulties—particularly during childhood—remains critical for timely intervention and favorable developmental outcomes.

In applied settings, multimethod integration may involve combining performance-based tests to assess cognitive efficiency, rating scales to capture everyday functioning, and—where feasible—computerized or neurobiological measures to enhance ecological and theoretical precision. The selection and integration of methods should be guided by assessment goals, developmental stage, and contextual demands.
