# Supplementary material for: Executive functions in research and practice: a multimethod review of behavioral, subjective, and neurobiological assessment tools
Source: Front Psychol. 2026 Mar 3;17:1713652. doi: 10.3389/fpsyg.2026.1713652 (PMC12992236; doi:10.3389/fpsyg.2026.1713652)
Supplement: Supplementary file 1 [file Table_1.docx]

**Supplementary Material A**

**Database Search Strings and Search Strategy**

This supplementary material provides the exact search strings and procedures used to identify studies for the narrative mini-review *“Executive Functions in Research and Practice: A Multimethod Review of Behavioral, Subjective, and Neurobiological Assessment Tools.”* The searches were conducted in accordance with the methodological description reported in the main manuscript.

**Databases Searched**

- Scopus
- Web of Science
- PubMed
- JSTOR
- Google Scholar
- HEAL-Link (Hellenic Academic Libraries Link)

**Search Period**

January 2000 – December 2024

**Search Terms and Boolean Logic**

Searches were conducted using combinations of keywords related to executive functions, assessment, and specific executive components. Minor adaptations were made to accommodate database-specific syntax and indexing requirements.

**Scopus & Web of Science**

("executive functions" OR "executive functioning")

AND (assessment OR measurement OR evaluation)

AND (inhibition OR "cognitive flexibility" OR updating OR planning)

Filters applied:

- Document type: Article, Review
- Language: English or Greek
- Subject areas: Psychology, Neuroscience, Education, Psychiatry

**PubMed**

("executive function"[Title/Abstract] OR "executive functions"[Title/Abstract])

AND (assessment OR measurement OR evaluation)

Additional filters:

- Humans
- Peer-reviewed journals
- Publication date: 2000–2024

**JSTOR**

"executive functions" AND assessment

Results were manually screened to exclude non-empirical papers and publications lacking methodological detail on assessment tools.

**Google Scholar**

"executive functions" AND assessment

AND (behavioral OR "rating scales" OR computerized OR neuroimaging)

The first 200 results sorted by relevance were screened, in line with common narrative review practices.

**HEAL-Link**

Searches were conducted using English and Greek terms, including:

"executive functions" AND assessment

εκτελεστικές λειτουργίες AND αξιολόγηση

**Additional Search Procedures**

- Reference lists of key reviews and empirical articles were manually screened to identify potentially relevant studies.
- No records were identified through organizational websites, trial registries, or citation tracking beyond database searches, consistent with the PRISMA flow diagram.

**Study Selection**

All retrieved records were screened based on titles and abstracts. Full-text screening was conducted for potentially eligible articles according to the inclusion and exclusion criteria described in the Methods section of the main manuscript. The final selection process is summarized in the PRISMA 2020 flow diagram (Figure 1).
